# Multi-Scale Multivariate Models for Small Area Health Survey Data: A Chilean Example

**DOI:** 10.3390/ijerph17051682

**Published:** 2020-03-05

**Authors:** Andrew Lawson, Anna Schritz, Luis Villarroel, Gloria A. Aguayo

**Affiliations:** 1 Department of Public Health Sciences, Medical University of South Carolina, Charleston, SC 29466, USA; 2 Luxembourg Institute of Health, 1A-B, rue Thomas Edison, Strassen, L-1445 Luxembourg City, Luxembourg; Anna.Schritz@lih.lu (A.S.); Gloria.Aguayo@lih.lu (G.A.A.); 3 Public Health Department, School of Medicine, Pontificia Universidad Católica de Chile, Diagonal Paraguay 362, Santiago 8330077, Chile; lv@med.puc.cl

**Keywords:** Bayesian modeling, multivariate, multi-scale, spatial correlation, sample weights

## Abstract

Background: We propose a general approach to the analysis of multivariate health outcome data where geo-coding at different spatial scales is available. We propose multiscale joint models which address the links between individual outcomes and also allow for correlation between areas. The models are highly novel in that they exploit survey data to provide multiscale estimates of the prevalences in small areas for a range of disease outcomes. Results The models incorporate both disease specific, and common disease spatially structured components. The multiple scales envisaged is where individual survey data is used to model regional prevalences or risks at an aggregate scale. This approach involves the use of survey weights as predictors within our Bayesian multivariate models. Missingness has to be addressed within these models and we use predictive inference which exploits the correlation between diseases to provide estimates of missing prevalances. The Case study we examine is from the National Health Survey of Chile where geocoding to Province level is available. In that survey, diabetes, Hypertension, obesity and elevated low-density cholesterol (LDL) are available but differential missingness requires that aggregation of estimates and also the assumption of smoothed sampling weights at the aggregate level. **Conclusions:** The methodology proposed is highly novel and flexibly handles multiple disease outcomes at individual and aggregated levels (i.e., multiscale joint models). The missingness mechanism adopted provides realistic estimates for inclusion in the aggregate model at Provincia level. The spatial structure of four diseases within Provincias has marked spatial differentiation, with diabetes and hypertension strongly clustered in central Provincias and obesity and LDL more clustered in the southern areas.

## 1. Introduction

Often, health outcome data arises where measures are made on individuals who reside within geographical regions of a country. A survey may have been carried out to obtain a ‘snapshot’ of the health of the study region. In addition, the survey could be useful in providing insight into prevalences of disease within aggregated regions of the study window. Recently, Vanderdijck et al., [1] proposed an approach to the modeling of small area health survey data by utilizing the survey weight as a predictor within a conventional generalized linear mixed model, thereby making allowance for the survey design aspect of the study. Watjou et al. [2] extended this approach to situations where there is non-response. Alternative approaches have been proposed whereby the outcome itself is adjusted via sample weights [3,4,5]. However, assuming a conventional generalized linear mixed model (GLMM) with an unadjusted outcome variable has a variety of advantages not least of which is that conventional software can be used for modeling and hence flexible extension to models is straightforward. Multi-scale models have also been developed where models allow borrowing of information across scales [6,7,8,9]. In the most recent examples, models at higher levels of spatial aggregation can inherit effects from finer resolution levels, individual-level models can inherit contextual effects from aggregate regions. In what follows, we assume individual level models for survey participants and aggregate level models for Provincia estimation. We assume that spatial effects can be captured using Markov Random Field (MRF) models as we have discrete spatial units [10]. 

The Chilean National Health Survey (CNHS) is a large population cross-sectional study representative of the adult Chilean population, which is performed every 6 years. The main objectives of the CNHS are to assess and monitor the prevalence of adult health problems in the general Chilean population and to describe its variation according to sex, age, socioeconomic level, rural-urban area and geographic region. This is to facilitate the planning and evaluation of preventive and curative strategies. The survey provides information about a range of diseases (physical and mental health) and risk factors. Data are collected by self-reported questionnaires, a physical examination visit and biological samples. In the CNHS, type 2 diabetes, hypertension, obesity and high cholesterol, amongst the major outcomes of the metabolic syndrome, are available. After 1980, with economic development, the prevalence of obesity and other chronic diseases continued to increase in Chile. In the period from 2003 to 2017, both type 2 diabetes and obesity prevalence showed marked increases in the CNHS surveys. 

One big challenge of the data collection of the CNHS was the particular Chilean geography with an uneven distribution of population across the country in a central-overcrowded and in remote, isolated and depopulated areas that were very difficult to access [11].

### 1.1. Objectives

Our focus is the modeling of multiple individual survey outcomes and the extension of this to a multi-scale approach to aggregate estimation of disease prevalence or risk. By multi scale, we mean analysis of more than one geographic resolution (scale) level. By aggregate, we mean that data are accumulated into larger geographic regions. This multi-scale objective extends the idea of small area estimation by jointly modeling different geo-scales at the same time adjusting for survey response and biases.

### 1.2. Data Background and Availability 

The aim of this study was to present a methodology that can be used in the analysis of multiple disease outcomes and that can provide combined estimates of prevalence at various spatial scales. The data we used are from a cross-sectional study of the 2009–2010 CNHS. The regions used in the analysis were 52 out of 54 provinces of Chile. The population in each province varies from a few thousand to over 4 million in the largest province Santiago. The sampling frame was constituted from the 2002 Population and Housing Census which was the most recent Census to the study period. The study design was based on a random sample of households of complex type (stratified and multistage by clusters) with national, regional and rural/urban representation. The target population was adults older than or equal to 15 years. The survey had a response rate in the eligible population of 85%. Our data consist of a total of 4780 individuals with differing patterns of missingness. The data from the survey consist of individual clinical, demographic, and behavioral variables for survey participants. Diabetes outcome was defined as fasting glucose ≥126 mg/dl or self-reported medical diagnosis (not during pregnancy). Obesity was defined as body mass index (BMI) HTA ≥ 30 kg/m^2^, hypertension was defined as systolic blood pressure ≥ 140 mmHg or diastolic blood pressure ≥ 90 mmHg or self- report of arterial hypertension (HTA) treatment. Elevated low density lipoprotein (LDL) was defined according to the cardiovascular adult treatment panel (ATP) III Update (> 100 mg/dl (if there is already cardiovascular disease), > 130 mg/dl (if moderate cardiovascular reactivity(CVR)) or > 160 mg/dl (if low CVR)). The 2009–010 CNHS included a total of 5293 individuals, although only 4780 participated in the two examinations and the laboratory test. All outcome definitions are based on the official report [12] and data provided by the CNHS. The demographic variables included from the survey were age, gender and their interaction. In addition, the individuals were geo-referenced to various spatial administrative units (such as regions, provinces and communes). In the following analysis, the province level was used throughout. The data are publicly available at http://epi.minsal.cl/bases-de-datos/.

The basic map of Chile and its provinces was taken from http://www.rulamahue.cl/. It has to be mentioned that the map only represents 52 provinces instead of the current 54. One reason is that Easter Island, which is counted as one province, is not on the map and was not included in the survey. The other missing province is Marga Marga, which was created in 2009 and became operative in 2010. Marga Marga consists of two communes of the Valparaiso province and two communes of the Quillota province. As the survey was carried out during the creation of the new province, it was decided to use the map that was still valid when the survey started.

**Ethics approval and consent to participate:** The data used in this study are public domain as part of CNHS and the analysis does not require ethical approval as individual cases are not labelled or referenced. Provincial level inference is made only. 

## 2. Methods

Denote a set of surveyed individual responses as $y_{ik},\;i=1,....,m;\;k=1,....,K$ where the *i* th person has kth outcome. The vector of outcomes for each person is denoted by $y_{i}$. The survey consists of *m* participants and the disease outcomes are defined as $y_{ik}\;i=1,\ldots ,m;\;m=4780$. We assume that this binary outcome for the k th disease is Bernoulli distributed as $y_{ik}∼Bern(p_{ik})$ and we modeled the logit of the probability of positive outcome. The logit consists of fixed and random effects to account for both observed confounding and unobserved outcome confounding. Hence, $\mathrm{logit}(p_{ik})=x_{i}^{t}\beta_{k}+z_{i}^{t}\gamma_{k}$ will be assumed where $x_{i}^{t}\beta_{k}$ is a linear predictor including observed confounders, and $z_{i}^{t}\gamma_{k}$ is a linear combination of random effects. As the individuals were sampled from within a population, we must include within our specification the sampling weight used for each person. Following the proposal in [1,2], we add the sampling weight ($sw_{i}$) as a fixed effect within the linear predictor.

The random effects included within the model were chosen to represent the different forms of unobserved effects. First, we assume an individual frailty effect could be present and add an uncorrelated disease specific individual level random effect $v_{ik}$. Next, we include spatial effects to represent the area within which the individual resides (province only). Two effects are estimated for the province level: an uncorrelated effect $v_{j_{\left(i\in j\right)}}^{p}$, where the subscript represents the *j* th province within which the *i* th person resides, and a spatially correlated effect $u_{j(i\in j)}^{p}$.

The overall model used for this analysis is a Bayesian formulation and so all parameters in the model have prior distributions, as follows: $v_{ik}∼N(0,\tau_{vk}^{-1});\;v_{j}^{p}∼N(0,\tau_{vp*}^{-1});\;u_{j}^{p}∼ICAR(\tau_{up}^{-1})$. The ICAR distribution is a special Markov random field prior distribution which includes spatial correlation (see, e.g., [13]; [10], ch. 5) and essentially captures the clustering tendency of the outcome via neighborhood adjacency. Note that this is a common assumption for the analysis of discrete small area spatial structure, rather than geostatistical models which require distance-based correlation to be specified [10]. The uncorrelated effects have zero mean Gaussian distribution with small precisions which should provide non-informativeness. There are 52 provinces in Chile, so that j=1,…,n where n=52 and Table A1 provides a basic summary of the regions.

### 2.1. Joint Scale Models

The overall model used for this analysis is a binary logistic model at the individual level for each of the *k* outcomes. A Bayesian formulation is assumed and so all parameters in the model have prior distributions, as follows:

$v_{ik}∼N(0,\tau_{vk}^{-1});\;v_{j}^{p}∼N(0,\tau_{vp}^{-1});\;u_{j}^{p}∼ICAR(\tau_{up}^{-1})$. The ICAR distribution is a special prior distribution with

(1) $$\begin{array}{l} u_{jk}^{p}|u_{l\neq j,k}^{p}∼N(\bar{u}{\bar{p}}_{\delta_{j}k},\tau_{up_{k}}^{-1}/n_{\delta_{j}})\;\mathrm{where}\;\bar{u}{\bar{p}}_{\delta_{j}k}=\sum_{l\in \delta_{j}}u_{{}_{l\neq j,k}}^{p}/n_{\delta_{j}}\;\\ \mathrm{and}\;\delta_{j}\;\mathrm{is}\;\mathrm{the}\;\mathrm{neighborhood}\;\mathrm{set}\;\mathrm{of}\;\mathrm{the}\;j\;\mathrm{th}\;\mathrm{location}\;\mathrm{with}\;n_{\delta_{j}}\;\mathrm{the}\;\mathrm{number}\;\mathrm{of}\;\mathrm{neighbors}. \end{array}$$

This can model spatial structure (see, e.g., Besag [13]; Lawson [10], chapter 5) and essentially assumes that neighboring areas are positively correlated. It is assumed that each outcome has a different variance of the spatial field and hence, the precisions have a *k* subscript. Note that while this ICAR formulation is dependent on neighborhood definition, it is an adaptive prior specification as the variance depends on the number of neighbors of any given region. This allows for edge regions to have larger variance and smaller precision. To estimate the probability of the diseases for each province, a binomial model on aggregated province data was used as in Aregay, Lawson, Faes, Kirby, Carroll and Watjou [8], Aregay, Lawson, Faes and Kirby [9]. The binomial model assumed here is an approximation and we assume that the random effect structure employed makes allowance for this misspecification. The outcome was defined as cases of each disease per province out of the number of individuals sampled per province. The mean sampling weight per province ($sw_{j}^{p}$), the mean age per province ($Age_{j}^{p}$), the percentage of male individuals per province ($Sex_{j}^{p}$) and their interaction were defined as adjusting variables. The same uncorrelated and correlated random spatial effects from the individual model were used in the aggregated model. This was done to use the spatial information gained from the individual analysis for the aggregated model. Hence, for the *k* th outcome, with age x sex adjustment,

(2) $$y_{ik}∼\;Bern\left(p_{ik}\right)\;individual\;level$$

(3) $$logit\left(p_{ik}\right)=\beta_{0k}+Age_{i}\;*\beta_{1k}+Sex_{i}*\;\beta_{2k}+Age_{i}*Sex_{i}*\;\beta_{3k}\;+f_{k}\left(sw_{i}\right)+v_{ik}+v_{jk}^{p}+u_{jk}^{p}$$

(4) $$y_{jk}^{p}∼\;Bin\left(p_{jk}^{p},\;n_{j}^{p}\right)\;province\;level$$

(5) $$logit\left(p_{jk}^{p}\right)=\;\alpha_{0k}+Age_{j}^{p}*\alpha_{1k}+Sex_{j}^{p}*\alpha +Age_{j}^{p}*Sex_{j}^{p}*\;\alpha_{3k}\;+f_{k}\left(sw_{j}^{p}\right)+v_{jk}^{p}+u_{jk}^{p}$$

where $n_{j}^{p}$ is the sample size in the $j^{th}$ province and $N_{j}^{p}$ is the population of the province. $p_{jk}^{p}$ is the probability of the outcome and $y_{jk}^{p}=\sum_{i\in j}y_{ik}$ where the sum is over all the individuals within the *j* th province. A crude unadjusted estimate of prevalence could be computed as $y_{jk}^{p}/n_{j}^{p}$. 

### 2.2. Missingness and Imputation

All missing data were imputed within our Bayesian models: outcomes were imputed using predictive distribution and predictors were imputed, where appropriate, from an assumed prior distribution. Any missing predictors were given suitable prior distributions and imputed within the Markov Chain Monte Carlo (McMC) algorithms. 

Some provinces had no sampling, in which case they must have their prevalence estimated. We did this as follows. Assume restricted prior distributions for

(6) $$n_{j}^{p}∼\;Pois\left(110\right)I\left(1,\right)$$

and

(7) $$p_{j}^{p}∼\;Beta\left(1,1\right)$$

and use the predictive distribution to yield

(8) $$y_{j}^{pm}<-bin\left(p_{j}^{p},\;n_{j}^{p}\right).$$

For missing sampling weights, the following estimation for province level sampling weights was performed. Denote the average sampling weight for a province as

(9) $$sw_{j}^{p}=\;\underset{l\;\in j}{\sum }sw_{l}/n_{j}^{p}$$

for provinces sampled and

(10) $$sw_{j}^{p}=\;\bar{sw}$$

where $\bar{sw}$ is the global mean sampling weight (for those areas sampled). Hence,

(11) $$sw_{j}^{p}=\;\bar{sw}+I_{j}\left(sw_{j}^{p}-\bar{sw}\right)$$

(12) $$I_{j}=\;\{\begin{array}{c} 1,\;non-missing\\ 0,\;missing \end{array}$$

So that the sampling weight for the missing areas is the global average.

### 2.3. Joint Outcome Modelling

While a multi-scale model is assumed for a given disease outcome, we also have a range of outcomes that are a focus of this study. Outcomes related to the metabolic syndrome were considered important to examine. These include diabetes, obesity, hypertension and elevated LDL. For each of these, a joint multi-scale model was assumed, but to allow linkage between the outcomes at the individual level, a joint model approach was implemented assuming that there is a shared random effect between the k=4 outcome variables. This means that models for each outcome on the individual data level and aggregated data level were run during the same Markov Chain Monte Carlo iterations. The individual data level models included a common random effect $vs_{i}$ for each individual. 

Individual Level Models (I1)

(13) $$\begin{array}{l} y_{ik}∼Bern(p_{ik})\\ \mathrm{logit}(p_{ik})=\beta_{0k}+\beta_{1k}Age_{i}+\beta_{2k}Sex_{i}+\beta_{3k}Age_{i}*Sex_{i}+\beta_{4k}sw_{i}+R_{kij}\;\mathrm{model}\;I1\\ \;\mathrm{and}\;R_{kij}=v_{ik}+vs_{ik}+v_{jk}^{p}+u_{jk}^{p}\\ with\\ v_{i*},v_{j*}^{p},vs_{i*},\beta_{*}∼N(0,\tau_{*}^{-1})\\ u_{jk}^{p}∼ICAR(\tau_{up_{k}}^{-1}). \end{array}$$

Note that the random effects are chosen to represent our belief that individuals vary independently but multiple measurements on an individual will have some commonality: Hence, $v_{ik}+vs_{i}$ is a composite effect. We also assume that individuals inherit a contextual effect of province and so $v_{jk}^{p}+u_{jk}^{p}$ are jointly modelled with the aggregate level.

Aggregate (Provincia) Level Models (A1)

At the provincial level, we approximate the model by assuming that the sampled count is

(14) $$\begin{array}{l} y_{jk}^{p}∼bin(p_{jk}^{p},n_{j}^{p})\;\forall_{k}\\ \mathrm{and}\\ \mathrm{logit}(p_{jk}^{p})=\\ \alpha_{0k}+\alpha_{1k}Age_{j}^{p}+\alpha_{2k}Sex_{j}^{p}+\alpha_{3k}Age_{j}^{p}*Sex_{j}^{p}+f_{k}(sw_{j}^{p})\\ +R_{jk}^{p}\;\mathrm{model}\;A1\\ where\;R_{jk}^{p}=v_{jk}^{p}+u_{jk}^{p}\;\forall_{k}\\ and\;f_{k}(sw_{j}^{p})=\gamma .sw_{j}^{p} \end{array}$$

where there are assumed to be different confounder effects for each outcome. This binomial approximation affects the variance of the aggregate count, but the inclusion of random effects at this level allows for the adjustment of variance. Note that there are eight models fitted jointly with some separate and shared random effects.

### 2.4. Model Fitting and Goodness of fit 

Model I1 and AI were fitted jointly for all disease outcomes. Posterior sampling via McMC was chosen as the main tool for estimation. Given that missingness occurs within the outcomes and predictors, we assumed a Bayesian paradigm and decided to use BUGS software as it allows the imputation of missing outcomes using data augmentation, and allows prior specification for missing predictors. All joint models were fitted using WinBUGS14 [14]. Maps of Chile were created using the tmap package in R [15]. Two chains were run with a thinning of 50. Samples of a size of 5000 were taken following burn-in (Lunn, et al. [16], Lunn, et al. [17]). Convergence was visually checked by means of Gelman–Rubin–Brooks plot (Brooks and Gelman [18]), the potential scale reduction factor $\hat{R}$ (Gelman and Rubin [19]), sample trace and density plots, sample autocorrelations and Markov Chain Monte Carlo error (Lawson, et al. [20]). Convergence can be assumed if $\hat{R}$ is close to 1. Model fit was visually checked by means of trace plots of the deviance (Spiegelhalter, et al. [21]), mapping the correlated heterogeneity, up, and uncorrelated heterogeneity, vp. The map of the correlated heterogeneity shows a clustered pattern and the map of uncorrelated heterogeneity shows a random pattern, if an adequate model fit is found. 

As is common in other cross-sectional studies the missingness in this study did not display any particular structure and was assumed to be essentially at random (MAR). This form of missingness is handled optimally using predictive inference within the Bayesian modeling framework. 

### 2.5. Posterior Risk Exceedance

As an additional diagnostic to help with the delineation of areas of exceptionally high risk, we employed exceedance probability criteria (Lawson [22]). An exceedance probability can be computed from posterior sampled output from a Bayesian model (see, e.g., Lawson and Rotejanaprasert [23]). The exceedance probability is defined as the probability that the estimated posterior value, in this case prevalence, for each sample, was greater than a chosen threshold.

(15) $$P\left(p_{kj}>c\right)=\;\frac{1}{G}\;\overset{G}{\underset{g=1}{\sum }}\;I(p_{kj}>c)$$

(16) $$I=\{\begin{array}{c} 1,\;p_{kj}>c\;\\ 0,\;otherwise\; \end{array}$$

where G is the total number of samples, p is the estimated probability of sample g and c is the chosen threshold. Whenever p exceeds the threshold c, it is recorded as 1, 0 otherwise. Averaged over the sample, this yields an estimate of the upper tail marginal probability of the parameter. This can be used to detect unusually high values and for hot spot clustering by looking for groups of unusually high areas in mapped output (Richardson, et al. [24]).

For diabetes, a threshold of 9.4%, for obesity of 25.1%, for hypertension of 26.9% and for elevated LDL a threshold of 22.7% was chosen based on the estimated national prevalence in the CNHS report (http://www.minsal.cl/estudios_encuestas_salud/). Exceedance results are reported in Table A1, Table A2, Table A3 and Table A4.

### 2.6. Descriptive Statistics

The mean age of the study population was 46.3 with no significant difference in age between males (45.7) and females (46.7). More females (59.9%), than males (40.1%) participated in the study (*p*-value < 0.001). Females showed a significantly higher BMI than males (28.1 vs. 27.4; *p*-value < 0.001). Women had a higher, but not significant, diabetes rate (10.7% vs. 10.4%; *p*-value = 0.8331), a significantly higher obesity rate (32.9% vs. 23.4%; *p*-value < 0.001), a lower hypertension rate (34.3% vs. 37.2%; *p*-value = 0.03895) and a lower significant elevated LDL rate (24.5% vs. 34.6%; *p*-value = < 0.001) than men.

**Figure 1:**
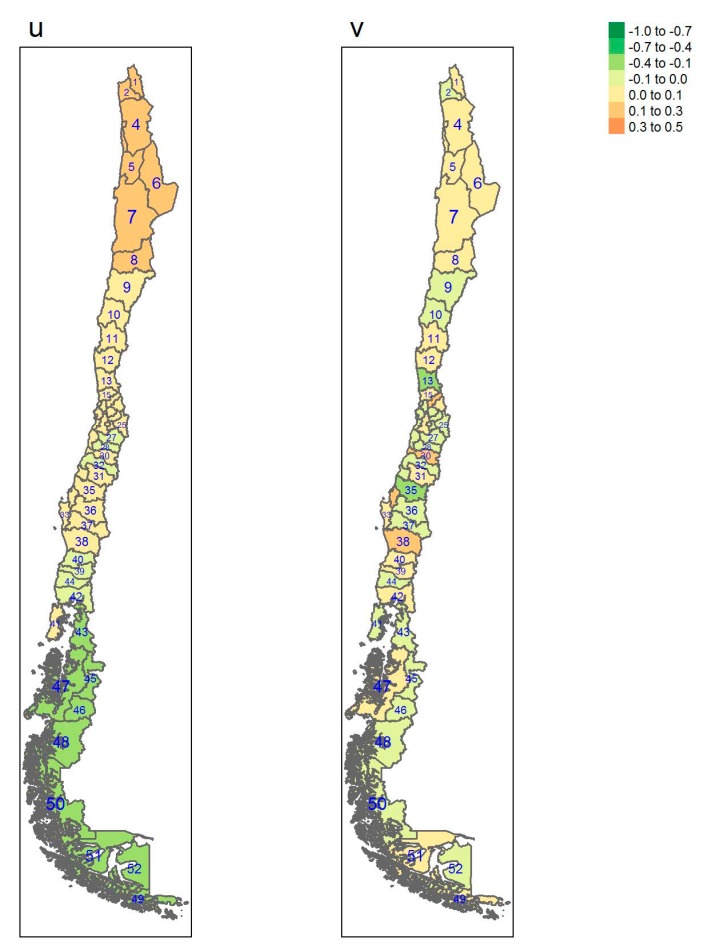
Multi-scale model heterogeneity effects for diabetes (u: correlated effect; v: uncorrelated effect).

**Figure 2:**
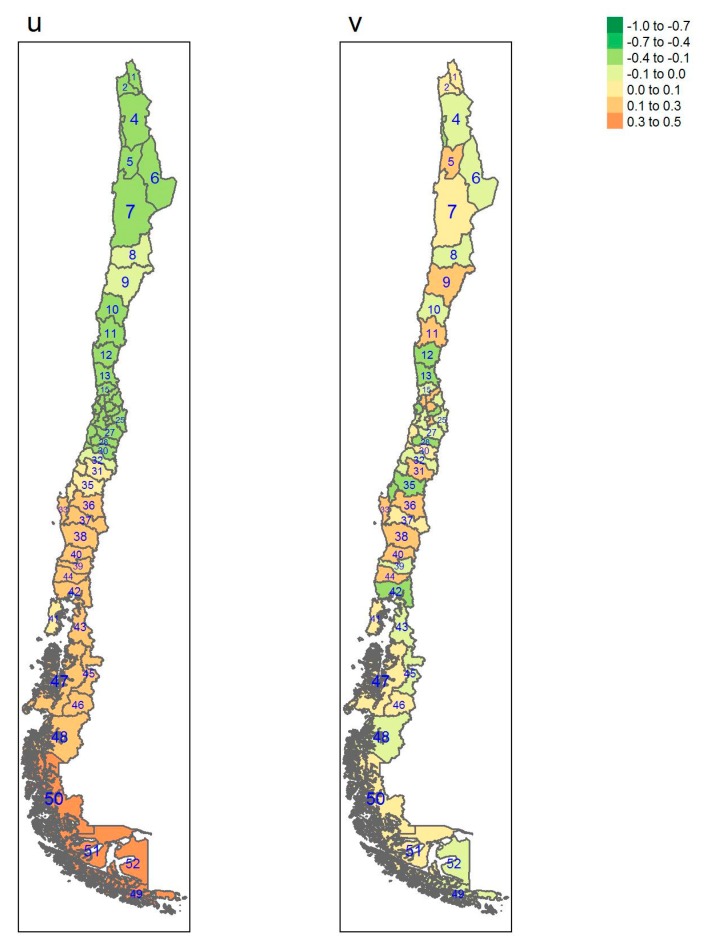
Multi-scale model heterogeneity effects for obesity (u: correlated effect; v: uncorrelated effect).

**Figure 3:**
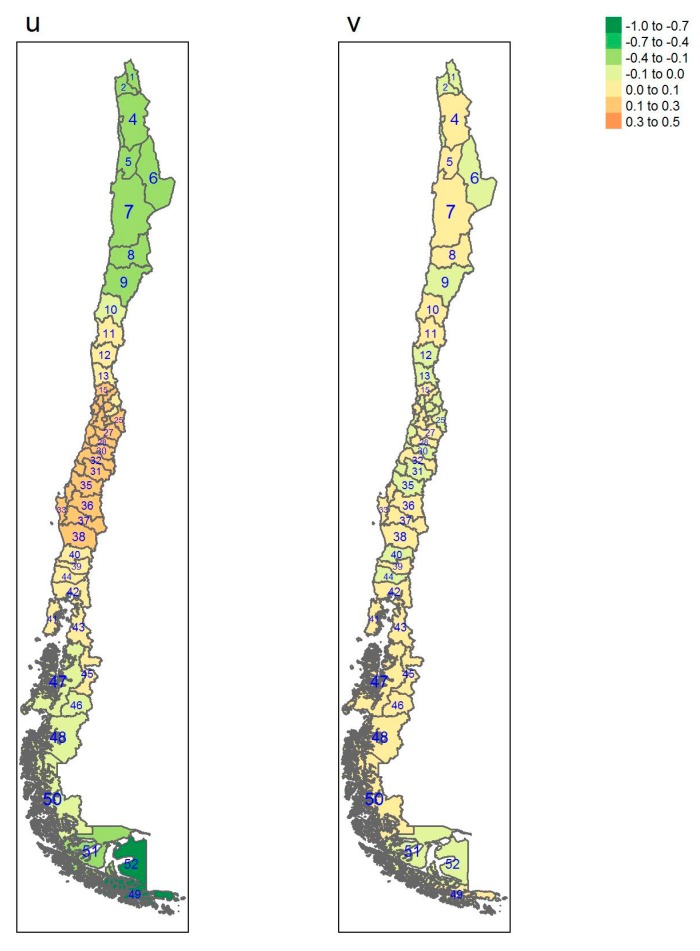
Multi-scale model heterogeneity effects for hypertension (u: correlated effect; v: uncorrelated effect).

**Figure 4:**
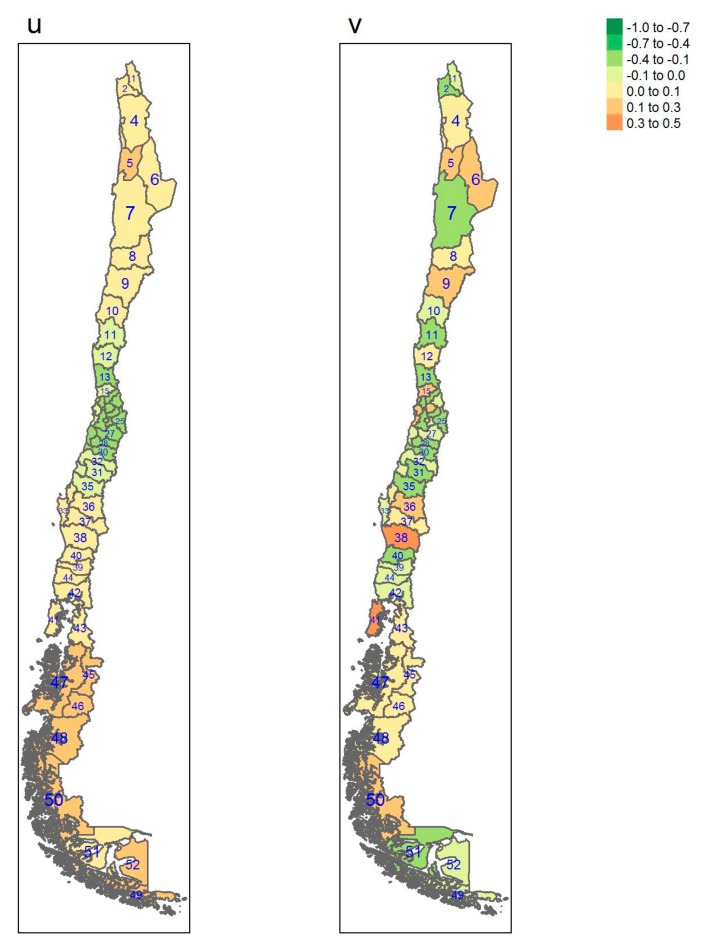
Multi-scale model heterogeneity effects for elevated LDL (u: correlated effect; v: uncorrelated effect).

Table 1 gives some information concerning which region each province belongs to, square km, Inhabitants per square km, gross domestic product (GDP) per capita and the number of sampled individuals per province. Table A1, Table A2 and Table A3 show estimates of posterior probabilities, their standard deviations (SD), median, 2.5% and 97.5% percentiles of the 95% credible interval, and the probability of exceeding the chosen threshold for each province and each outcome. In Figure 1, Figure 2, Figure 3 and Figure 4, posterior estimates of a range of quantities for the fitted spatial models for diabetes, hypertension, obesity and elevated LDL are found. 

## 3. Results of the Joint Modeling

For Diabetes (Table A1), Cauquenes (29) had the highest posterior probability of diabetes, with 16.27%. Provinces with a 95% or higher probability of exceeding the threshold of 9.4% of diabetes were Limari (12), San Felipe de Aconcagua (19), Melipilla (22), Colchagua (28), Cauquenes (29), Curicó (30), Concepción (34) and Cautín (38). Those diabetes hotspots are all located in the central part of Chile. Table A2 provides the results for the analysis of obesity. The highest posterior probability of obesity was estimated in Antártica Chilena (49) with 40.60%, but it did not appear to be significantly higher than the chosen threshold of 25.1% and the estimate actually mainly depends on spatial prior distributions as no samples were taken in Antártica Chilena (49) itself. The highest posterior probability was for Magallanes (51) with 38.52%. Obesity hotspots are mostly found in the central to southern part of Chile. In Table A2, posterior probabilities of hypertension per province are listed. The highest probability of hypertension was found in Cauquenes (29) with 57.14%. Hypertension hotspots are mostly found in the central to southern part of Chile. Table A4 demonstrates that the highest probability of elevated LDL level was estimated in Ultima Esperanza (50) with 41.79%, though estimation relies on assumed prior distributions as no samples were observed in this province itself. The highest exceedance probability was for Copiaco (9), 37.15%, Cautin (38) 40.06%, and Chiloe (41) 40.28% which all have a 100% exceedance of the national rate.

Overall, it would appear that diabetes and hypertension display a similar distribution in that there are concentrations of both in central metropolitan areas and also in Southern Chile. Obesity, on the other hand, is less marked in central area but shows elevation on the south also. Cholesterol (elevated LDL) demonstrates a similar pattern to obesity in terms of the north–south gradient, but displays less prevalence in central areas and is overall less clustered.

In the joint analyses reported here, it is clear that diabetes and hypertension display a similar spatial distribution of risk in that central and southern areas are the most affected and exceedance probabilities > 0.95 are commonly found. In contrast, elevated LDL and obesity are more marked in the southern parts of the country and show fewer examples of exceedance than for diabetes or hypertension. This suggests a more uniform pattern of risk for elevated LDL over the country than for the other outcomes.

Figure 1, Figure 2, Figure 3 and Figure 4 display the multi-scale model heterogeneity effects for each outcome.

It is notable that for all the outcomes the uncorrelated effects are relatively random, whereas the correlated effects display distinct clustering. For diabetes, the clustering is marked in the northern regions, whereas the clustering is marked in the south for obesity. Hypertension displays a clustering in the central regions whereas elevated LDL shows clustering in the most southerly areas of the Magallanes region, with lower central area effects.

### Linear Model Parameter Estimates

Table 2 displays the posterior mean estimates for the predictors included in each of the individual and aggregate models for the four outcomes. Some general features of the analysis should be highlighted. First, in the aggregate models, only the intercept and spatial random effects were well estimated. The aggregate survey weights were not well estimated for any outcome, and neither were the age and gender predictors. For the individual level outcomes, different effects emerged. In all the outcomes, the sampling weight was not found to be well estimated. The intercept was well estimated, as was age for all outcomes. Age was found to be positively related to diabetes, obesity, cholesterol and hypertension. Differences arise in the effect of gender and the age x gender interaction. While gender and the age x gender interaction were not well estimated for diabetes, there is a well estimated negative effect of gender (male) on obesity, and a positive effect for cholesterol and hypertension. In addition, while most age x gender interactions were not well estimated, it was found well estimated for hypertension. 

Although hypertension tends to display similar spatial patterning to diabetes, there are marked differences in the individual predictors associated with each outcome. Hypertension has a gender and age x gender association that is not seen in diabetes.

## 4. Discussion

There are some notable correlations and disparities between the distributions of diabetes, hypertension, obesity and elevated LDL. Both diabetes and hypertension have marked prevalences in the central provinces, including the metropolitan areas around Santiago. Note that while marked differences arise between outcomes, at the individual level, we have included a person-specific and outcome-specific individual effect. This leaves some allowance for correlation between outcomes in the individual. At the province level, we did not include shared province effects, but individuals had multi-scale sharing of province effects. The regression parameter posterior estimates demonstrate that while similar spatial patterning can arise, there are also differences at the individual level that can be marked. The central regions elevation of diabetes and hypertension could be explained by the urbanicity of the areas around Santiago and the associated lifestyle trends. The concentration of obesity and elevated LDL in the southern regions may reflect differentials with northern comparison regions and in particular dietary practices. 

In this example, the sampling weight appears to have little impact on any outcome whether at individual or aggregate level. That said, it is important to include the sampling weight as it represents factors affecting the inclusion of participants in the survey.

## 5. Conclusions

The joint analysis of these four metabolic outcomes demonstrates the benefit of considering the correlation between outcomes at the individual level. It also demonstrates the benefit of a multi-scale analysis in that individual outcomes can be modelled contextually within provinces and they can inherit the grouping effect of the province. In addition, the inclusion of survey weights at different levels is an important feature that allows the analysis to proceed, taking into consideration sampling effects. The joint analysis allowed the estimation of prevalences at the aggregate level while also providing contextual effects for the individuals. We did not assume here that the province level outcomes should share effects but in future work, we could explore sharing further especially for diabetes and hypertension (e.g., shared spatial effects), which appear to have similar patterning.

## Figures and Tables

**Table 1:** Characteristics of provinces and regions in Chile (^a^ Data obtained from Clark-Núñez X. Compendio estadístico. Instituto Nacional de Estadísticas Chile, 2017).

Regions	Area (km^2^) ^a^	Inhabitants/km^2^ ^a^	GDP/Capita USD ^a^	Provinces (Number)	Sampled
**North**					
Arica y Parinacota	16,873	14.6	9848	Parinacota (1)	0
				Arica (2)	311
Tarapacá	42,226	8.4	27,604	Iquique (3)	289
				Tamarugal (4)	24
Antofagasta	126,049	5.1	63,402	Tocopilla (5)	34
				El Loa (6)	88
				Antofagasta (7)	183
Atacama	75,176	4.3	27,882	Chañaral (8)	0
				Copiapó (9)	226
				Huasco (10)	81
Coquimbo	40,580	19.6	14,800	Elqui (11)	185
				Limarí (12)	72
				Choapa (13)	49
**Center**					
Valparaíso	16,396	113.4	17,009	San Antonio (14)	34
				Petorca (15)	17
				Valparaíso (16)	187
				Quillota (17)	48
				Los Andes (18)	25
				San Felipe de Aconcagua (19)	34
Metropolitana	15,403	485.8	24,224	Chacabuco (20)	13
				Santiago (21)	728
				Melipilla (22)	26
				Talagante (23)	18
				Maipo (24)	49
				Cordillera (25)	77
O’Higgins	16,387	57	17,985	Cardenal Caro (26)	0
				Cachapoal (27)	211
				Colchagua (28)	102
Maule	30,296	34.9	10,620	Cauquenes (29)	19
				Curicó (30)	85
				Linares (31)	108
				Talca (32)	139
Biobío	37,069	57.8	12,582	Arauco (33)	51
				Concepción (34)	134
				Ñuble (35)	57
				Biobío (36)	49
**South**					
Araucanía	31,842	31.5	8,376	Malleco (37)	67
				Cautín (38)	261
Los Ríos	18,430	22.3	11,711	Ranco (39)	71
				Valdivia (40)	228
Los Lagos	48,584	17.6	13,335	Chiloé (41)	74
				Llanquihue (42)	151
				Palena (43)	0
				Osorno (44)	92
**Far South**					
Aysén	108,494	1	19,851	Coyhaique (45)	185
				General Carrera (46)	98
				Aysén (47)	0
				Capitan Prat (48)	0
Magallanes	1,382,291	0.1	18,447	Antártica Chilena (49)	0
				Última Esperanza (50)	56
				Magallanes (51)	243
				Tierra del Fuego (52)	14

**Table 2:** Posterior mean estimates for regression parameters in the joint model for diabetes, obesity, cholesterol (elevated LDL), and hypertension for both individual and aggregate level models.

Disease Outcome	Model	Parameters	Mean	SD	2.50%	97.50%
**Diabetes**	Individual level model	Intercept	−4.088	0.508	−5.242	−3.222
		Survey weight	0.000	0.000	0.000	0.000
		Age	0.081	0.011	0.063	0.105
		Sex male	−0.027	0.149	−0.348	0.266
		Age * Sex male	0.006	0.009	−0.010	0.023
	Aggregated model (per province)	Intercept	−2.365	0.336	−3.063	−1.722
		Mean Survey weight	0.000	0.000	0.000	0.000
		Mean Age	0.097	0.098	−0.077	0.303
		Proportion Male	0.375	0.791	−1.161	2.051
		Mean age * proportion male	−0.112	0.231	−0.599	0.304
**Obesity**	Individual level model	Intercept	−1.182	0.174	−1.557	−0.894
		Survey weight	0.000	0.000	0.000	0.000
		Age	0.029	0.005	0.020	0.041
		Sex male	−0.793	0.148	−1.111	−0.536
		Age * Sex male	−0.005	0.006	−0.018	0.006
	Aggregated model (per province)	Intercept	−0.826	0.281	−1.364	−0.224
		Mean Survey weight	0.000	0.000	0.000	0.000
		Mean Age	−0.008	0.068	−0.148	0.129
		Proportion Male	−0.142	0.665	−1.567	1.142
		Mean age * proportion male	0.011	0.159	-0.310	0.345
**Cholesterol**	Individual level model	Intercept	−2.410	0.330	−3.145	−1.839
		Survey weight	0.000	0.000	0.000	0.000
		Age	0.102	0.014	0.078	0.134
		Sex male	1.118	0.227	0.709	1.608
		Age * Sex male	−0.005	0.010	−0.025	0.013
	Aggregated model (per province)	Intercept	−0.895	0.340	−1.586	−0.204
		Mean Survey weight	0.000	0.000	0.000	0.000
		Mean Age	0.063	0.090	−0.109	0.261
		Proportion Male	0.144	0.812	−1.517	1.813
		Mean age * proportion male	−0.043	0.213	−0.511	0.363
**Hypertension**	Individual level model	Intercept	−1.542	0.137	−1.848	−1.313
		Survey weight	0.000	0.000	0.000	0.000
		Age	0.142	0.010	0.127	0.168
		Sex male	0.501	0.118	0.274	0.734
		Age * Sex male	−0.022	0.007	−0.037	−0.008
	Aggregated model (per province)	Intercept	−0.890	0.253	−1.396	−0.424
		Mean Survey weight	0.000	0.000	0.000	0.000
		Mean Age	0.052	0.063	−0.071	0.183
		Proportion Male	0.742	0.612	−0.345	1.982
		Mean age * proportion male	0.044	0.149	−0.268	0.339

* Interaction.

## Appendix A

**Table A1:** Diabetes.

Region	Nr	Province	Mean	Sd	2.50%	97.50%	>= 9.4%
**Arica y Parinacota**	1	Parinacota	13.12%	6.36%	4.67%	29.04%	74.09%
	2	Arica	10.91%	1.33%	8.42%	13.65%	87.87%
**Tarapaca**	3	Iquique	11.23%	1.37%	8.78%	14.14%	91.99%
	4	Tamarugal	12.17%	2.53%	8.24%	18.18%	90.05%
**Antofagasta**	5	Tocopilla	10.36%	1.95%	7.04%	14.92%	67.75%
	6	El Loa	9.03%	1.77%	5.98%	12.97%	38.03%
	7	Antofagasta	10.56%	1.48%	7.98%	13.82%	77.99%
**Atacama**	8	Chanaral	12.72%	6.13%	4.39%	28.23%	72.01%
	9	Copiapo	10.33%	1.42%	7.61%	13.22%	74.90%
	10	Huasco	11.90%	1.96%	8.20%	16.08%	90.33%
**Coquimbo**	11	Elqui	10.51%	1.49%	8.00%	13.87%	76.80%
	12	Limari	12.81%	2.14%	9.26%	17.70%	97.02%
	13	Choapa	9.70%	1.78%	6.11%	13.20%	57.78%
**Valparaiso**	14	San Antonio	9.37%	2.09%	5.59%	13.89%	47.25%
	15	Petorca	13.02%	3.01%	8.29%	20.00%	92.05%
	16	Valparaiso	11.53%	1.51%	8.64%	14.54%	92.25%
	17	Quillota	12.39%	1.99%	8.74%	16.75%	94.13%
	18	Los Andes	10.97%	2.13%	7.42%	15.88%	78.08%
	19	San Felipe de Aconcagua	15.45%	3.35%	10.61%	23.80%	99.63%
**Metropolitana**	20	Chacabuco	6.23%	1.94%	3.02%	10.53%	6.24%
	21	Santiago	9.87%	0.96%	8.08%	11.79%	68.00%
	22	Melipilla	14.23%	3.19%	9.28%	21.94%	97.19%
	23	Talagante	11.19%	5.14%	3.85%	23.94%	57.26%
	24	Maipo	10.19%	1.83%	6.79%	14.12%	66.56%
	25	Cordillera	8.73%	1.61%	5.81%	12.23%	31.43%
**O’Higgins**	26	Cardenal Caro	11.54%	5.79%	4.14%	25.96%	62.77%
	27	Cachapoal	10.64%	1.34%	8.05%	13.28%	81.97%
	28	Colchagua	12.96%	2.15%	9.01%	17.50%	95.99%
**Maule**	29	Cauquenes	16.27%	3.73%	9.83%	24.52%	98.30%
	30	Curico	13.72%	2.27%	10.18%	18.95%	99.31%
	31	Linares	9.80%	1.53%	7.10%	13.16%	58.38%
	32	Talca	7.79%	1.35%	5.16%	10.51%	11.15%
**Biobio**	33	Arauco	11.94%	2.07%	8.55%	16.68%	92.13%
	34	Concepcion	13.03%	2.19%	9.56%	18.02%	98.15%
	35	Nuble	8.67%	1.93%	5.04%	12.58%	34.00%
	36	Biobio	10.97%	2.01%	6.94%	14.96%	78.73%
**Araucania**	37	Malleco	12.32%	2.70%	7.52%	18.01%	86.75%
	38	Cautin	12.10%	1.46%	9.61%	15.30%	98.49%
**Rios**	39	Ranco	11.17%	1.78%	7.93%	15.09%	85.17%
	40	Valdivia	10.78%	1.41%	8.19%	13.83%	83.66%
**Lagos**	41	Chiloe	9.87%	1.64%	6.50%	13.04%	63.11%
	42	Llanquihue	10.84%	1.66%	7.77%	14.39%	81.12%
	43	Palena	10.32%	5.19%	3.60%	22.95%	50.33%
	44	Osorno	10.30%	1.67%	7.16%	13.85%	70.61%
**Aysen**	45	Coyhaique	7.89%	1.40%	5.30%	10.79%	13.88%
	46	General Carrera	5.84%	1.70%	3.02%	9.65%	3.24%
	47	Aisen	10.20%	5.04%	3.50%	22.88%	49.23%
	48	Capitan Prat	10.24%	5.30%	3.49%	23.05%	48.79%
**Magallanes**	49	Antartica Chilena	11.53%	10.18%	0.79%	38.62%	46.47%
	50	Ultima Esperanza	10.37%	2.18%	6.46%	14.92%	66.17%
	51	Magallanes	10.14%	1.45%	7.49%	13.20%	68.81%
	52	Tierra del Fuego	10.02%	7.59%	0.93%	30.10%	43.23%

**Table A2:** Obesity.

Region	Nr	Province	Mean	Sd	2.50%	97.5%	>= 25.1%
**Arica y Parinacota**	1	Parinacota	26.78%	8.04%	12.72%	44.93%	55.31%
	2	Arica	27.56%	2.21%	23.31%	32.03%	86.87%
**Tarapaca**	3	Iquique	20.13%	2.10%	16.15%	24.31%	1.10%
	4	Tamarugal	25.16%	4.51%	17.05%	34.94%	48.28%
**Antofagasta**	5	Tocopilla	29.75%	4.57%	21.69%	39.63%	85.25%
	6	El Loa	26.58%	3.60%	19.96%	33.88%	64.71%
	7	Antofagasta	29.27%	2.67%	24.19%	34.61%	94.48%
**Atacama**	8	Chanaral	28.05%	8.11%	13.66%	45.79%	62.97%
	9	Copiapo	31.07%	2.59%	26.04%	36.32%	99.06%
	10	Huasco	25.55%	3.48%	19.02%	32.66%	53.45%
**Coquimbo**	11	Elqui	31.02%	2.83%	25.63%	36.85%	98.51%
	12	Limari	19.42%	3.29%	13.28%	26.08%	4.38%
	13	Choapa	21.32%	3.72%	14.27%	28.89%	15.34%
**Valparaiso**	14	San Antonio	25.54%	4.50%	17.32%	35.01%	51.71%
	15	Petorca	25.98%	5.24%	16.52%	36.87%	54.40%
	16	Valparaiso	24.07%	2.47%	19.36%	29.06%	32.85%
	17	Quillota	28.66%	4.16%	21.21%	37.69%	80.42%
	18	Los Andes	25.62%	4.40%	17.56%	35.02%	52.64%
	19	San Felipe de Aconcagua	31.03%	5.26%	21.90%	42.40%	87.65%
**Metropolitana**	20	Chacabuco	31.00%	6.38%	19.88%	44.79%	82.27%
	21	Santiago	23.76%	1.50%	20.91%	26.74%	18.82%
	22	Melipilla	25.59%	4.65%	17.09%	35.46%	52.14%
	23	Talagante	28.56%	7.58%	15.11%	44.83%	65.43%
	24	Maipo	31.41%	4.72%	23.19%	41.38%	92.34%
	25	Cordillera	25.57%	3.46%	19.09%	32.72%	54.29%
**O’Higgins**	26	Cardenal Caro	26.95%	7.71%	12.97%	44.19%	57.30%
	27	Cachapoal	25.51%	2.34%	21.08%	30.35%	55.86%
	28	Colchagua	20.36%	3.04%	14.65%	26.59%	6.44%
**Maule**	29	Cauquenes	27.40%	5.54%	17.43%	38.99%	64.62%
	30	Curico	28.48%	3.60%	21.76%	35.87%	82.54%
	31	Linares	35.18%	3.67%	28.25%	42.72%	99.86%
	32	Talca	29.70%	3.14%	23.79%	36.13%	93.31%
**Biobio**	33	Arauco	36.04%	4.40%	27.91%	45.27%	99.70%
	34	Concepcion	28.41%	3.18%	22.42%	34.85%	85.14%
	35	Nuble	27.06%	4.38%	18.77%	35.99%	66.45%
	36	Biobio	38.28%	4.88%	29.40%	48.44%	99.83%
**Araucania**	37	Malleco	32.71%	4.50%	24.41%	41.91%	96.12%
	38	Cautin	36.05%	2.52%	31.22%	40.95%	100.0%
**Rios**	39	Ranco	33.18%	3.94%	25.66%	41.02%	98.29%
	40	Valdivia	36.70%	2.77%	31.39%	42.25%	100.0%
**Lagos**	41	Chiloe	31.38%	3.60%	24.77%	38.92%	96.85%
	42	Llanquihue	29.43%	3.10%	23.42%	35.65%	92.21%
	43	Palena	34.23%	8.99%	17.55%	53.47%	86.27%
	44	Osorno	37.03%	3.82%	29.84%	44.70%	99.96%
**Aysen**	45	Coyhaique	32.59%	2.98%	26.81%	38.54%	99.61%
	46	General Carrera	37.74%	4.47%	29.34%	46.83%	99.87%
	47	Aisen	35.06%	9.11%	17.98%	54.83%	88.13%
	48	Capitan Prat	35.46%	9.02%	18.53%	54.36%	88.76%
**Magallanes**	49	Antartica Chilena	40.60%	16.12%	12.75%	74.23%	81.67%
	50	Ultima Esperanza	36.02%	4.80%	26.72%	45.82%	99.06%
	51	Magallanes	38.52%	2.66%	33.37%	43.79%	100.0%
	52	Tierra del Fuego	39.59%	12.42%	17.18%	64.96%	87.56%

**Table A3:** Hypertension.

Region	Nr	Province	Mean	Sd	2.50%	97.5%	>= 6.9%
**Arica y Parinacota**	1	Parinacota	30.98%	10.7%	14.0%	56.98%	60.55%
	2	Arica	26.29%	2.07%	22.2%	30.28%	37.92%
**Tarapaca**	3	Iquique	28.26%	2.07%	24.3%	32.43%	74.40%
	4	Tamarugal	28.99%	3.56%	22.7%	36.87%	71.65%
**Antofagasta**	5	Tocopilla	27.65%	3.22%	22.1%	34.94%	55.83%
	6	El Loa	21.82%	2.70%	16.8%	27.56%	4.16%
	7	Antofagasta	27.59%	2.30%	23.3%	32.35%	60.34%
**Atacama**	8	Chanaral	33.29%	10.6%	16.1%	57.77%	71.07%
	9	Copiapo	31.35%	2.40%	26.7%	36.16%	96.99%
	10	Huasco	37.98%	3.34%	31.4%	44.58%	99.99%
**Coquimbo**	11	Elqui	32.75%	2.57%	27.9%	38.02%	99.27%
	12	Limari	39.20%	3.38%	32.6%	45.94%	100.0%
	13	Choapa	34.48%	3.50%	27.3%	41.25%	98.05%
**Valparaiso**	14	San Antonio	32.69%	3.89%	25.1%	40.47%	93.46%
	15	Petorca	43.10%	4.78%	34.1%	53.06%	99.99%
	16	Valparaiso	43.42%	2.59%	38.4%	48.70%	100.0%
	17	Quillota	44.17%	3.43%	37.2%	50.79%	100.0%
	18	Los Andes	34.27%	3.60%	26.8%	41.02%	97.45%
	19	San Felipe de Aconcagua	49.71%	4.01%	42.3%	58.24%	100.0%
**Metropolitana**	20	Chacabuco	19.94%	3.75%	13.4%	28.16%	4.25%
	21	Santiago	32.11%	1.63%	28.9%	35.36%	99.96%
	22	Melipilla	44.93%	4.53%	36.6%	54.68%	100.0%
	23	Talagante	24.46%	6.65%	13.2%	39.20%	32.86%
	24	Maipo	35.08%	3.28%	29.0%	42.06%	99.65%
	25	Cordillera	28.95%	2.90%	23.4%	34.86%	76.37%
**O’Higgins**	26	Cardenal Caro	40.45%	11.4%	21.1%	66.95%	90.67%
	27	Cachapoal	42.41%	2.37%	37.9%	47.25%	100.0%
	28	Colchagua	54.27%	3.40%	47.6%	60.89%	100.0%
**Maule**	29	Cauquenes	57.14%	5.03%	46.6%	66.67%	100.0%
	30	Curico	43.80%	3.21%	37.2%	49.89%	100.0%
	31	Linares	35.05%	2.95%	29.1%	40.84%	99.65%
	32	Talca	33.00%	2.79%	27.7%	38.66%	98.82%
**Biobio**	33	Arauco	42.95%	3.49%	36.3%	49.99%	100.0%
	34	Concepcion	39.90%	2.91%	34.3%	45.73%	100.0%
	35	Nuble	31.93%	3.45%	25.4%	38.97%	93.32%
	36	Biobio	45.97%	3.84%	39.1%	54.16%	100.0%
**Araucania**	37	Malleco	55.48%	4.28%	46.9%	63.95%	100.0%
	38	Cautin	41.86%	2.30%	37.4%	46.44%	100.0%
**Rios**	39	Ranco	43.22%	3.35%	36.9%	50.03%	100.0%
	40	Valdivia	36.74%	2.57%	31.7%	41.80%	99.99%
**Lagos**	41	Chiloe	37.79%	3.07%	32.5%	44.80%	100.0%
	42	Llanquihue	40.20%	2.95%	34.4%	46.03%	100.0%
	43	Palena	37.63%	10.9%	18.8%	62.70%	85.39%
	44	Osorno	41.31%	3.20%	34.9%	47.55%	100.0%
**Aysen**	45	Coyhaique	35.13%	2.75%	29.9%	40.73%	99.94%
	46	General Carrera	25.98%	3.66%	19.2%	33.50%	38.48%
	47	Aisen	37.11%	11.0%	18.6%	62.63%	83.58%
	48	Capitan Prat	36.50%	11.0%	17.9%	61.74%	81.35%
**Magallanes**	49	Antartica Chilena	21.46%	13.1%	3.92%	53.47%	28.10%
	50	Ultima Esperanza	44.71%	4.14%	36.7%	53.02%	100.0%
	51	Magallanes	34.74%	2.53%	29.8%	39.73%	99.94%
	52	Tierra del Fuego	17.54%	9.04%	4.24%	39.02%	15.05%

**Table A4:** Elevated LDL.

Region	Nr	Province	Mean	Sd	2.50%	97.50%	>= 22.7%
**Arica y Parinacota**	1	Parinacota	31.28%	11.00%	13.0%	57.03%	79.12%
	2	Arica	26.55%	2.95%	20.9%	32.58%	90.67%
**Tarapaca**	3	Iquique	32.05%	3.08%	26.1%	38.25%	99.94%
	4	Tamarugal	32.24%	6.37%	20.6%	45.62%	94.34%
**Antofagasta**	5	Tocopilla	35.08%	6.60%	23.9%	50.18%	98.60%
	6	El Loa	28.04%	5.06%	18.9%	38.90%	85.45%
	7	Antofagasta	25.39%	3.32%	19.0%	31.97%	78.74%
**Atacama**	8	Chanaral	31.46%	10.89%	13.1%	56.28%	79.48%
	9	Copiapo	37.15%	3.73%	30.1%	44.60%	100.00%
	10	Huasco	32.22%	4.73%	23.2%	41.95%	98.29%
**Coquimbo**	11	Elqui	23.41%	3.46%	16.9%	30.50%	57.46%
	12	Limari	31.65%	5.11%	22.1%	42.35%	96.66%
	13	Choapa	24.43%	4.70%	15.7%	33.96%	63.39%
**Valparaiso**	14	San Antonio	27.66%	6.25%	17.0%	41.47%	78.12%
	15	Petorca	29.60%	6.42%	18.4%	43.65%	86.55%
	16	Valparaiso	32.69%	4.26%	24.8%	41.49%	99.49%
	17	Quillota	27.66%	5.62%	17.1%	39.04%	81.23%
	18	Los Andes	24.60%	5.60%	14.4%	36.82%	61.88%
	19	San Felipe de Aconcagua	28.63%	5.91%	17.4%	40.53%	84.15%
**Metropolitana**	20	Chacabuco	18.36%	6.19%	8.67%	32.94%	21.41%
	21	Santiago	21.62%	1.92%	18.0%	25.52%	28.35%
	22	Melipilla	22.94%	5.86%	12.1%	35.33%	49.74%
	23	Talagante	17.94%	7.82%	6.16%	36.79%	23.61%
	24	Maipo	21.72%	4.56%	13.5%	31.52%	38.96%
	25	Cordillera	17.19%	3.93%	10.1%	25.48%	8.82%
**O’Higgins**	26	Cardenal Caro	27.64%	10.16%	11.1%	51.62%	67.34%
	27	Cachapoal	27.54%	3.51%	20.9%	34.74%	91.92%
	28	Colchagua	28.15%	4.70%	19.2%	37.80%	87.74%
**Maule**	29	Cauquenes	38.11%	7.30%	24.3%	53.16%	98.61%
	30	Curico	28.09%	4.27%	20.0%	36.80%	90.05%
	31	Linares	23.97%	3.90%	16.6%	31.96%	62.41%
	32	Talca	23.57%	3.28%	17.5%	30.35%	59.41%
**Biobio**	33	Arauco	29.18%	4.61%	20.5%	38.68%	92.55%
	34	Concepcion	28.95%	4.01%	21.5%	37.29%	94.71%
	35	Nuble	18.49%	4.61%	10.2%	28.28%	17.57%
	36	Biobio	33.47%	5.73%	23.3%	45.66%	98.26%
**Araucania**	37	Malleco	37.51%	5.83%	26.5%	49.18%	99.74%
	38	Cautin	40.06%	3.78%	32.8%	47.69%	100.00%
**Rios**	39	Ranco	32.08%	4.64%	23.3%	41.45%	98.33%
	40	Valdivia	26.19%	3.29%	20.0%	32.74%	85.61%
**Lagos**	41	Chiloe	40.28%	5.84%	29.8%	52.48%	100.00%
	42	Llanquihue	32.40%	3.76%	25.3%	39.86%	99.63%
	43	Palena	31.80%	10.87%	13.3%	56.29%	80.81%
	44	Osorno	30.01%	4.40%	21.6%	39.08%	95.58%
**Aysen**	45	Coyhaique	32.14%	4.34%	24.0%	41.11%	98.94%
	46	General Carrera	27.71%	4.70%	19.1%	37.26%	85.61%
	47	Aisen	32.16%	11.16%	13.3%	58.58%	81.17%
	48	Capitan Prat	32.20%	11.28%	13.3%	58.78%	81.19%
**Magallanes**	49	Antartica Chilena	37.17%	18.31%	7.79%	76.28%	75.29%
	50	Ultima Esperanza	41.79%	6.27%	30.2%	54.65%	99.96%
	51	Magallanes	31.45%	3.41%	24.9%	38.19%	99.74%
	52	Tierra del Fuego	34.33%	13.49%	11.5%	62.79%	78.95%
